# Enhancing work life through social cognition: the effect of SCIT in bipolar disorder

**DOI:** 10.3389/fpsyg.2024.1470191

**Published:** 2025-01-30

**Authors:** Zeynep Anaforoglu Bikmaz, Zeynep Mackali, Sibel Cakir

**Affiliations:** ^1^Department of Psychology, Istinye University, Istanbul, Türkiye; ^2^Department of Psychology, Istanbul Bilgi University, Istanbul, Türkiye; ^3^Department of Psychiatry, Bahcesehir Medical School, Istanbul, Türkiye

**Keywords:** bipolar disorder, social cognition and interaction therapy (SCIT), occupational functioning, cognitive functioning, resistance to stigmatization

## Abstract

**Objectives:**

This study aimed to investigate the effectiveness of social cognition and interaction therapy (SCIT) in improving occupational functioning in individuals diagnosed with bipolar disorder I (BD-I). The effects of SCIT intervention with standard treatment methods were investigated on social cognition and functioning effects, which are often negatively affected in patients with BD-I.

**Methods:**

The research involved 28 participants, allocated into two groups: An experimental group (*n* = 12) receiving SCIT for 8 weeks alongside standard treatments (pharmacotherapy and psychiatric interviews), and a control group (*n* = 16) undergoing standard treatment without additional interventions. Outcome measures were assessed using a suite of tools, including the Hamilton Depression Rating Scale (HDRS), Young Mania Rating Scale (YMRS), Eyes Test (ET), Metacognition Scale (MCI), Internalized Stigma of Mental Illnesses (ISMI), and Functioning Assessment Short Test (FAST). Given the small sample size, non-parametric tests were employed for data analysis. Analyses were conducted using the Mann–Whitney *U* test and Wilcoxon test for comparisons between the experimental and control groups and within groups. Also, the effects of the intervention on social cognition, occupational functioning and resistance to stigmatization were dealt.

**Results:**

The findings revealed that participants in the experimental group showed significant improvements in social cognition and occupational functioning after SCIT compared to the control group. However, the levels of stigmatization experienced by individuals as a result of ISMI measurements were significantly lower in the SCIT group compared to the control group.

**Conclusion:**

The study concludes that SCIT can be an effective intervention for enhancing certain psychosocial and cognitive functions in individuals with BD-I, thereby improving their occupational functioning. Nevertheless, the persistent levels of stigma indicate the need for additional strategies to address the broader challenges faced by individuals with BD-I in terms of societal perception and self-stigmatization.

## 1 Introduction

Bipolar disorder (BD) is a mood disorder with periods of mania, hypomania and depression that deeply affects the individual’s family, work and social life. Functioning, which is the ability to meet one’s basic needs and establish relationships with others, can cause many negative effects in the lives of individuals diagnosed with bipolar disorder during episodes. One of the areas negatively affected in terms of functionality is occupational functionality. Occupational functioning is the ability of individuals to perform their profession or job effectively, to fulfill their authorities and responsibilities, and to establish harmonious relationships with the people they work with in the work environment (Frank et al., 2008). Individuals diagnosed with bipolar disorder may be absent from the workplace for long periods of time during episodes. Following manic and depressive periods, these patients are monitored during remission. While the medication treatment of patients continues, decreases in areas of functionality necessitate different interventions (Bowden, 2005). In studies aimed at enhancing the efficiency of functionality areas (such as family life, work, and social relationships, etc.) for individuals diagnosed with BD, it has been observed that they struggle to adapt to interpersonal relationships and social life, along with stigma and neurocognitive impairments (Zarate et al., 2000).

The prevalence and recurrence rates of BD are significant for the social and economic impacts it has and can have (Abdul Pari et al., 2014). Evaluation of workdays lost due to BD- related issues reveals that bipolar disorder ranks second among causes of job loss and is among the top five most common reasons for work impairment when considering work-disruptive situations. Additionally, long-term unemployment and absenteeism related to BD have been found to be associated with low job performance (Bowden, 2005; Dean et al., 2004; Grande et al., 2013; Sylvia et al., 2017; Martinez-Camarillo et al., 2019).

Individuals with BD, like in other mental health conditions, require certain specialized treatment and support mechanisms (such as psychotherapy, psychoeducation) in addition to medication treatment to strengthen functionality areas (Sylvia et al., 2015). The most crucial reason for the need to support functionality in the workplace is the recurrence of episodes in cases where work and family life are not supported in the illness picture (Simon et al., 2008). The support of functionality in the workplace is also crucial for individuals diagnosed with BD to meet their economic and social needs, continue their treatments, and cope with societal stigma (Sylvia et al., 2017). Among the problems frequently experienced by individuals diagnosed with BD in the workplace, issues such as stigma, sleep disturbances, attention deficits, difficulty coping with work stress, inability to manage interpersonal relationships, and difficulties in regulating emotions are prominent (Martinez-Camarillo et al., 2019).

Social cognition can be considered as an important mechanism that influences interpersonal relationships in terms of beliefs, desires, emotions, and thoughts. It has been noted that social cognition, which is also considered as one of the working areas of the brain, does not function adequately in individuals diagnosed with BD, regardless of symptoms. This condition may be one of the underlying reasons for difficulties in social relationships, exacerbation of illness symptoms, and triggering of episodes (Gillissie et al., 2022). Working on social cognition in psychotherapy helps individuals diagnosed with BD, who experience difficulties in focusing on their own and others’ mental states, to regulate their emotions and thoughts related to these situations (Kerr et al., 2003).

The first clinical studies on social cognition were conducted by Penn and colleagues in 2005 with schizophrenia patients in a hospital in the United States. The adaptation of the program for bipolar disorder was implemented in 2013 following its success (Penn et al., 2005; Lahera et al., 2013). Tas et al. (2012) conducted a study where the concepts of social cognition and mentalization were shared through concrete and visual examples from the workplace. This study provides an opportunity within a structured and well-defined framework. Social cognition and interaction training (SCIT), a group therapy technique that can also be conducted as individual therapy, was initially implemented in hospitals as a 15-week program. This study was developed with specific goals in mind and the program duration was shortened to 8 weeks. SCIT sessions focusing on work life in BD are planned for 8 weeks, with sessions held weekly, consisting of two 40-min sessions with a 10-min break, totaling 90 min (Zhang et al., 2019).

In a study evaluating the role of social cognition in the global functioning of optimally bipolar patients along with other relevant clinical variables and general cognitive measurements, it was concluded that social cognition could play a significant role in the clinical-functional differences of bipolar patients (Lahera et al., 2013). Another study conducted with schizophrenia in remission found that SCIT could be an applicable and promising method for improving social cognition and social functioning (Wang et al., 2013). In another study examining the effectiveness of SCIT among individuals with severe mental illness, SCIT plus social mentoring provides preliminary evidence that SCIT improves social cognition and functioning among individuals with severe mental illness living in the community (Hasson-Ohayon et al., 2014). Significant positive developments in patients’ life skills were found with SCIT in individuals with schizophrenia spectrum disorder receiving care through a public mental health service (Gordon et al., 2018). Another study investigating the effectiveness of family-focused therapies on individuals diagnosed with BD found that SCIT reduced cognitive distortions, increased cognitive flexibility, facilitated understanding of people’s perspectives in social communication by changing cognitive biases, and consequently led to reduced internalized stigma. Additionally, it was noted that SCIT reduced cognitive distortions in perceiving and understanding emotions in social relationships and provided a broader perspective on communication issues, particularly within the family (Tabas et al., 2023).

When examining the research conducted within the scope of SCIT from its inception in 2005 to the present day, it is observed that it has been widely applied not only with schizophrenia spectrum patients but also with those diagnosed with BD and depression. Particularly in the areas of social cognition and functionality, effective results have been achieved in patients undergoing SCIT (Penn et al., 2005; Lahera et al., 2013; Hasson-Ohayon et al., 2014; Gordon et al., 2018). Within the scope of this study, the effectiveness of SCIT in improving occupational functioning in work life in people diagnosed with BD type I was investigated. For this purpose, a significant increase in the levels of occupational functioning and social cognition and a significant decrease in stigmatization in the intervention group were hypothesized. As far as we can see from the literature, there is a limited number of studies examining the effectiveness of SCIT in improving occupational functioning in individuals diagnosed with BD-I. In this context, our study, which aims to improve the occupational functioning of patients with BD-I through SCIT, is a research issue of unique value. This study will provide a basic perspective for the development of similar studies and studies in different areas of functioning.

## 2 Methods

### 2.1 Participants

This study was conducted in the Mood Disorder Outpatient Clinic of the Department of Mental Health and Diseases, Istanbul University Faculty of Medicine between December 2018 and June 2019. Patients aged 20–45 years with a diagnosis of bipolar disorder type I according to DSM-V were included in the study. Other criteria were that the patients should be in remission for at least 3 months, have at least primary school education, have a work experience and participate voluntarily. Patients with any neurologic disease, intellectual disability, additional diagnoses of alcohol or substance abuse, and patients who had received electro convulsive treatment (ECT) within the last 6 months were excluded from the study. Patients were allowed to take some mood stabilizers medications. All participants continued their regular medication regimen and remained under psychiatric follow-up. Therefore, the effect of medication use was not considered a variable in the study, and it was assumed to be evenly distributed across both groups. Given that all participants adhered to their regular medication treatment and were under psychiatric supervision, the impact of medication use was not evaluated as a variable in the research. The study was approved by the Ethics Committee of Istanbul Arel University. Individuals diagnosed with BD-I based on DSM-5 diagnostic criteria, who were in a remission period and able to participate regularly, were included in the study. Participants who had undergone electroconvulsive therapy (ECT) or had severe comorbid psychiatric disorders were excluded. These criteria were established to ensure a more precise evaluation of the effectiveness of SCIT in individuals with bipolar disorder.

### 2.2 Procedure

The 32 participants who voluntarily agreed to participate in the study were randomly assigned to two groups as intervention and control groups. In the intervention group, 16 participants received SCIT in addition to standard treatment (medication + follow-up). Since the recommended number of participants for group therapy was 8, two separate intervention groups of 8 participants each were conducted at different times, taking into account the days convenient for the participants (Zhang et al., 2019). The control group consisted of 16 participants who received only standard treatment (medication + psychiatric interview). Prior to the intervention study, the Informed Consent Form was shared with the participants. The demografic information form prepared for the participants who agreed to consent was filled out in a face-to-face interview lasting approximately 1 h. Sociodemographic data on age, gender, marital status, education level, functionality, duration of illness, traumatic experience, and other treatments received by the participants were obtained in individual interviews conducted before the study. Then, the application of the scales determined for the research continued. The scales were applied to the participants in the same order. Scale application took approximately 60 min. At the end of the 8-week implementation, individual process evaluation interviews were conducted with the participants who completed the intervention.

### 2.3 Measures

The Hamilton Depression Rating Scale (HDRS) consisting of 17 items and the Young Mania Rating Scale (YMRS) were used to determine the depression and hypomania/mania levels of the participants. Being in remission in terms of bipolar disorder was determined as ≤6 for depression and ≤8 for mania, considering the last 3 months (Samalin et al., 2016; Jensen et al., 2016). The items related to difficulty falling asleep, waking up in the middle of the night, waking up early in the morning, somatic and sexual symptoms, weight loss and insight were graded 0–2 and the other items were graded 0–4. A maximum of 53 points can be obtained from the scale. In the evaluation of the HDRS-17 scale; 0–7 points indicate no depression, 8–15 points indicate mild depression, 16–28 points indicate moderate depression and 29 points and above indicate severe depression (Akdemir et al., 2001). The YMRS is used to determine the severity of the momentary manic state, not to make a diagnosis. A score of 12 points and above was determined as a marker of manic episode (Karadağ et al., 2002). The last 1 week was evaluated for manic episodes.

The Functioning Assessment Short Test (FAST) was used to assess individuals’ psychosocial functioning and occupational functioning. The Turkish version of FAST consists of 24 items and six dimensions. The FAST scale, comprising 24 items that assess various dimensions of functionality, is rated on a scale from 0 (no difficulty) to 3 (severe difficulty), requiring individuals to evaluate the extent of challenges they face in each domain. The scores from all subdomains are aggregated to calculate the overall level of functionality, with higher scores indicating more significant functional impairments. The dimensions of the scale are autonomy, occupational functioning, cognitive functioning, financial issues, interpersonal relationships and leisure time activities. In reliability analyses, Cronbach’s alpha internal consistency coefficient was 0.91 in the original version of the scale, while it was 0.96 in our measurements (Rosa et al., 2007; Aydemir and Uykur, 2012). The Eyes Test (ET), developed by Baron-Cohen et al. (1997) to assess emotion recognition skills, was used to assess social cognition. The ET consists of 36 pictures showing only the facial contour of the participants. Participants are asked to choose the emotion that best describes the mental state of the person among the four options shown to them (Baron-Cohen et al., 1997).

The Metacognition Scale (MCI) was used for detailed assessment of cognitive capacity. Each item in the MCI, which assesses how aware the person is of his/her mental processes, is answered on a 4-point Likert-type rating scale with endpoints of “(1) strongly disagree” and “(4) strongly agree.” The scores that can be obtained from the scale vary between 30 and 120 and a high score indicates an increase in pathological metacognitive activity (Tosun and Irak, 2008).

The Internalized Stigma Scale of Mental Illness (ISMI) was applied to measure internalized stigma. The scale, developed by Ritsher and Phelan (2004), consists of 29 items and has five subscales: alienation, confirmation of stereotypes, perceived discrimination, social withdrawal, resistance to stigmatization. Cronbach’s alpha coefficient of the scale was determined as 0.93. There is no cut-off score and it has been reported that internalized stigma increases as the score increases (Ersoy and Varan, 2007).

### 2.4 Design and intervention procedure

The study was planned as an experimental design with randomized control group. A total of 28 patients diagnosed with BD-I, 12 for the intervention group and 16 for the control group, participated in the study. Individual interviews with all participants were audio recorded before and after the intervention with one-hour interviews. The data of four participants in the intervention groups who did not complete the post-tests and the 8 week program were not included in the statistical evaluation. Individual interviews with all participants were audio and written recorded with one-hour interviews before and after the intervention. SCIT intervention was carried out regularly every week with the help of the SCIT-trained researcher ZB and a psychologist with clinical experience. It was conducted with the supervision of experienced clinical psychologist ZM. The Cronbach alpha values of HDRS, YMRS, FAST, MCI and ISMI measures are 0.75, 0.79, 0.96, 0.86, 0.93, respectively, and the reliability of the measures shows a good fit.

### 2.5 SCIT intervention

Developed to eliminate social cognitive deficits in people with psychotic disorders, SCIT is a manualized therapeutic technique (Penn et al., 2005). According to a study conducted in Finland, people with psychotic disorders can benefit from the modified SCIT program and find it feasible (Voutilainen et al., 2016). In the study, the suitability of the SCIT protocol was maintained and the 15-week program was modified to 8 weeks. The original SCIT protocol spans 15 weeks; however, this study implemented an 8-week format to mitigate transportation challenges and enhance participant retention. Considering the potential obstacles posed by hospital accessibility, a shorter program was deemed more practical for ensuring consistent attendance. Additionally, evidence from the literature supports the effectiveness of abbreviated SCIT protocols, demonstrating their capability to achieve comparable therapeutic outcomes (Zhang et al., 2019). The modified SCIT was applied in Turkish patients with BD-I. The intervention group, which consisted of two groups in total, received SCIT 1 day a week for 8 weeks. Each session lasted 90 min. The first 40 min of the sessions included providing information about the structured topic of the session and detailed examination of the case studies. The second 40 min of the sessions consisted of the participants’ personal experiences, problems and feedback. There was a 10 min break between both sessions. SCIT sessions consist of three stages: understanding emotions (two sessions), making situation assessment (three sessions) and gaining integration skills (three sessions). The weekly topics of the SCIT sessions are shown in Table 1.

**Table 1:** Eight-week SCIT session topics.

Week	Topic
1	Introduction, emotions and social situations
2	Describing and predicting emotions
3	Doubtful emotions
4	Jumping to conclusion
5	Generating other predictions
6	Separating facts from predictions
7	Gathering and integrating more evidence
8	Integration

### 2.6 Statistical analysis

SPSS 21.0 IBM was used to evaluate the data obtained. In the evaluation of the normality distribution of the data, Kolmogorov–Smirnov and Shapiro–Wilk analyzes were applied since the number of samples was less than 35. In the evaluation of data that did not show normal distribution, Mann–Whitney *U* tests were used for comparisons between two groups and Wilcoxon tests were used for within group evaluations. Demographic data were analyzed using descriptive statistics such as percentage, mean, and standard deviation. In the statistical analysis of the study, the results were accepted as 95% confidence interval and significance level was accepted as *p* < 0.05. The use of the reliable change index (RCI) analysis was deemed unsuitable for this study due to the non-parametric nature of the data. RCI is specifically designed for normally distributed continuous data and cannot be directly applied to ordinal or non-parametric datasets. Instead, non-parametric methods such as the Wilcoxon signed-rank test and Mann–Whitney *U* test were employed, offering an appropriate framework to analyze changes within and between the SCIT and control groups. Furthermore, effect sizes for non-parametric tests were calculated following established methodologies (Fritz et al., 2012), providing a robust and valid assessment of intervention effects. These approaches ensure reliable and accurate analyses for non-parametric datasets.

## 3 Results

### 3.1 Demographic characteristics of the patients

In the study, 28 volunteer participants with BD type I were randomly divided into experimental and control groups. Patients in the 12-person experimental group received SCIT in addition to standard treatment (medication + follow-up), while the 16-person control group received standard treatment (medication + psychiatric interview). Demographic properties of the participants between SCIT and control groups are presented in Table 2. There was no statistically significant difference between SCIT and control group in terms of each of the variables (*p* > 0.05).

**Table 2:** Demographic and distributional characteristics of SCIT and control group patients.

Items	SCIT (*n* = 12) mean (SD)	Control (*n* = 16) mean (SD)	Total (*n* = 28) mean (SD)	$\frac{\chi^{2}}{t}$	*p* ^*^
Gender (female)	5 (41.7%)	11 (68.8%)	16 (57.1%)	2.054	0.152
Gender (male)	7 (58.3%)	5 (31.2%)	12 (42.9%)		
Age (year)	30.75 (6.30)	31.12 (6.89)	30.96 (6.52)	97.50	0.945
Education level					
High school	4 (33.3%)	2 (12.5%)	6 (21.4%)	1.768	0.184
University & more	8 (66.7%)	14 (587.5%)	22 (78.6%)		
Employment status					
Employed	4 (33.3%)	7 (43.8%)	11 (39.3%)	0.312	0.576
Unemployed	8 (66.7%)	9 (56.2%)	17 (60.7%)		
Marital status					
Married	1 (8.3%)	6 (37.5%)	7 (25.0%)	3.111	0.078
Single	11 (91.7%)	10 (62.5%)	21 (75.0%)		
Psychiatric illness in family					
Yes	5 (41.7%)	7 (43.8%)	12 (42.9%)	0.012	0.912
No	7 (58.3%)	9 (56.2%)	16 (57.1%)		
Diagnosis supplement					
Yes	2 (16.7%)	0 (0.0%)	2 (7.1%)	2.872	0.090
No	10 (83.3%)	16 (100.0%)	26 (92.9%)		
Disease onset					
15–24 years	10 (83.3%)	8 (50.0%)	18 (64.3%)	3.319	0.069
25–40 years	2 (16.7%)	8 (50.0%)	10 (35.7%)		
Disease duration					
<5 years	6 (50.0%)	9 (56.2%)	15 (53.6%)	0.108	0.743
>5 years	6 (50.0%)	7 (43.8%)	15 (53.6%)		
Suicide attempt					
Yes	3 (25.0%)	6 (37.5%)	9 (32.1%)	0.491	0.912
No	9 (75.0%)	10 (62.5%)	19 (67.9%)		
Number of depression episodes					
<3	6 (50.0%)	10 (62.5%)	16 (57.1%)	0.438	0.508
>3	6 (50.0%)	6 (37.5%)	16 (57.1%)		
Number of manic episodes					
<3	9 (75.0%)	13 (81.2%)	22 (78.6%)	0.159	0.690
>3	3 (25.0%)	3 (18.8%)	6 (21.4%)		
Mixed episode					
Yes	4 (33.3%)	2 (12.5%)	6 (21.4%)	1.768	0.184
No	8 (66.7%)	14 (87.5%)	22 (78.6%)		
Hypothyroidism					
Yes	5 (41.7%)	2 (12.5%)	7 (25.0%)	3.111	0.078
No	7 (58.3%)	14 (87.5%)	21 (75.0%)		
ECT					
Yes	4 (33.3%)	2 (12.5%)	6 (21.4%)	1.768	0.184
No	8 (66.7%)	14 (87.5%)	22 (78.6%)		
Alcohol use					
Yes	3 (25.0%)	7 (43.8%)	10 (35.7%)	1.050	0.306
No	9 (75.0%)	9 (56.2%)	18 (64.3%)		
Frequency of alcohol use					
Less than 1 per month	10 (83.3%)	13 (81.2%)	23 (82.1%)	0.020	0.887
1 per week or more often	2 (16.7%)	3 (18.8%)	5 (17.9%)		
Cigarette use					
Yes	5 (41.7%)	9 (56.2%)	14 (50.0%)	0.583	0.445
No	7 (58.3%)	7 (43.8%)	14 (50.0%)		

*p<0.05: Statistically significant at the 95% confidence level.

### 3.2 Comparison of clinical variables between groups at baseline and post-intervention

When the normality of the pre- and post-test data was tested, the results were not normally distributed for both groups. Therefore, pre- and post-test data were analyzed using non- parametric tests. Using the Mann–Whitney *U* test, we compared clinical measures of HDRS, YMRS, ET, MCI, FAST and its six domains, ISMI and its five domains between SCIT and control group. There was no difference between the groups in the measures for both groups before the intervention. The analysis of clinical variables for the pre- and post-measurement data between SCIT and control group is presented in Table 2 (*p* values <0.05).

### 3.3 Comparison of clinical variables between groups at baseline and post-intervention

We calculated the eight-week pre- and post-intervention data of the SCIT group using the Wilcoxon test. For post-intervention data, the *p*-values for HDRS and ET were 0.003 and 0.006, respectively. There were no group differences in the total scores of the YMRS, MCI and ISMI. Our results showed that there were significant differences in ET and total FAST. Post-intervention tests showed that the total FAST score was significantly reduced in the SCIT group compared to those in the control group. Again, we analyzed changes in the sub-domains of the FAST. Pre- and post-intervention tests showed significant differences in the domains of occupational functioning, cognitive functioning, financial issues, interpersonal relationship and leisure time. Initial assessments of participants were conducted using the Hamilton Depression Rating Scale (HDRS), Young Mania Rating Scale (YMRS), Eyes Test (ET), Metacognition Scale (MCI), Functioning Assessment Short Test (FAST), and Internalized Stigma of Mental Illnesses (ISMI). The results revealed no significant differences between the experimental and control groups at the initial stage. However, post-SCIT intervention, the experimental group exhibited significant improvements in social cognition and occupational functioning (see Table 3).

**Table 3:** Clinical variables comparisons between intervention and control groups at baseline.

Items	SCIT (*n* = 12) mean (SD)	Control (*n* = 16) mean (SD)	*t*	*p* ^*^
HDRS	5.50 (5.47)	6.25 (4.17)	117.00	0.347
YMRS	2.17 (3.01)	1.00 (1.09)	79.00	0.450
ET	21.25 (3.44)	21.37 (3.56)	93.50	0.909
MCI	71.50 (13.13)	67.25 (12.68)	81.50	0.507
FAST total	38.50 (10.61)	37.37 (16.45)	95.50	0.982
^*^Autonomy	3.41 (2.23)	5.19 (3.90)	123.50	0.205
^*^Occupational functioning	9.67 (3.50)	8.75 (4.27)	83.00	0.568
^*^Cognitive functioning	2.75 (2.09)	2.75 (3.17)	81.00	0.507
^*^Financial issues	9.75 (3.98)	9.31 (5.00)	93.00	0.909
^*^Interpersonal relationship	9.92 (4.62)	8.00 (4.94)	72.50	0.280
^*^Leisure time	3.00 (1.28)	3.37 (1.67)	99.50	0.873
ISMI total	60.17 (12.48)	61.81 (10.12)	21.00	0.507
^*^Confirmation of stereotypes	12.17 (3.74)	11.62 (2.60)	83.00	0.568
^*^Perceived discrimination	10.58 (2.68)	10.56 (2.55)	92.50	0.873
^*^Alienation	13.17 (3.54)	13.06 (4.30)	88.50	0.732
^*^Social withdrawal	12.33 (4.42)	12.44 (4.00)	98.00	0.945
^*^Resistance to stigmatization	11.33 (1.82)	13.56 (2.66)	145.50	0.020

HDRS, Hamilton Depression Rating Scale; YMRS, Young Mania Rating Scale; ET, Eyes Test; MCI, Metacognition Scale; FAST, Functioning Assessment Short Test; ISMI, Internalized Stigma of Mental Illnesses.

When within- group interaction was taken into account, we found that the SCIT group showed superiority over the control group in all subdomains of the FAST. On the other hand, the resistance to stigmatization variable in the sub-domain of ISMI was significantly lower in the SCIT group compared to the control group (see Table 4).

**Table 4:** Comparison of all parameters within and between groups.

Items	Pre-SCIT (*n* = 12)	Post-SCIT (*n* = 12)	Pre-control (*n* = 16)	Post-control (*n* = 16)	Z_1_, p^*^ (within SCIT group)	Z_2_, p^*^ (within control group)	*t*, p^*^ (between group)
HDRS	5.50 (5.47)	2.00 (2.73)	6.25 (4.17)	5.69 (3.77)	0.00, 0.003	46.50, 0.441	109.00, 0.568
YMRS	2.17 (3.01)	1.25 (1.76)	1.00 (1.09)	0.94 (0.93)	3.00, 0.114	31.00, 0.854	115.00, 0.397
ET	21.25 (3.44)	24.50 (1.24)	21.37 (3.56)	20.87 (3.09)	64.00, 0.006	7.00, 0.057	85.50, 0.631
MCI	71.50 (13.13)	67.42 (12.96)	67.25 (12.68)	68.12 (11.65)	12.50, 0.068	98.50, 0.111	155.50, 0.004
FAST total	38.50 (10.61)	8.50 (8.56)	37.37 (16.45)	37.37 (15.13)	0.00, 0.002	42.00, 0.803	192.00, <0.001
^*^Autonomy	3.41 (2.23)	0.75 (1.21)	5.19 (3.90)	4.87 (3.54)	0.00, 0.011	7.000, 0.206	139.00, 0.047
^*^Occupational functioning	9.67 (3.50)	2.08 (2.87)	8.75 (4.27)	8.81 (4.04)	0.00, 0.002	15.50, 0.792	189.50, <0.001
^*^Cognitive functioning	9.75 (3.98)	2.00 (2.45)	9.31 (5.00)	9.31 (4.78)	0.00, 0.002	4.00, 0.713	191.50, <0.001
^*^Financial issues	2.75 (2.09)	0.92 (1.73)	2.75 (3.17)	2.87 (3.07)	10.50, 0.044	4.50, 0.414	139.00, 0.047
^*^Interpersonal relationship	9.92 (4.62)	2.00 (2.21)	8.00 (4.94)	8.12 (4.70)	1.00, 0.003	7.50, 0.317	176.00, <0.001
^*^Leisure time	3.00 (1.28)	0.75 (0.75)	3.37 (1.67)	3.37 (1.86)	0.00, 0.003	5.00, 1.000	181.00, <0.001
ISMI total	60.17 (12.48)	59.17 (11.49)	61.81 (10.12)	59.69 (8.52)	21.00, 0.507	13.50, 0.014	85.50, 0.631
^*^Confirmation of stereotypes	12.17 (3.74)	11.58 (4.19)	11.62 (2.60)	11.68 (3.07)	20.00, 0.440	30.00, 0.782	113.50, 0.423
^*^Perceived discrimination	10.58 (2.68)	10.58 (2.68)	10.56 (2.55)	10.69 (2.62)	10.50, 0.082	20.50, 0.723	130.50, 0.110
^*^Alienation	13.17 (3.54)	12.17 (3.81)	13.06 (4.30)	12.31 (3.70)	19.50, 0.225	2.50, 0.015	102.50, 0.797
^*^Social Withdrawal	12.33 (4.42)	11.58 (3.75)	12.44 (4.00)	11.50 (3.56)	15.50, 0.404	0.00, 0.004	92.50, 0.873
^*^Resistance to stigmatization	11.33 (1.82)	14.75 (2.96)	13.56 (2.66)	13.50 (2.92)	63.00, 0.007	25.00, 0.791	25.00, 0.001

HDRS, Hamilton Depression Rating Scale; YMRS, Young Mania Rating Scale; ET, Eyes Test; MCI, Metacognition Scale; FAST, Functioning Assessment Short Test; ISMI, Internalized Stigma of Mental Illnesses; Z_1_, comparison between the SCIT groups; Z_2_, comparison between the control groups. *p <0.01: Statistically significant at the 99% confidence level.

## 4 Discussion

The aim of this study was to evaluate the effectiveness of social cognition and interaction therapy (SCIT) as an additional intervention to standard treatments in individuals diagnosed with BD-I. After 8 weeks of psychosocial treatment of BD-I patients in randomly assigned SCIT and control groups The aim of this study was to evaluate the effectiveness of SCIT as an adjunct intervention to standard treatments for individuals diagnosed with BD-I. Over an 8-week psychosocial treatment period, BD-I patients were assigned to either SCIT or control groups through randomization, which was conducted using a computer-based software to ensure equal allocation probabilities. During this process, researchers and evaluators were blinded to the group assignments, minimizing the risk of evaluator bias. Analysis of the findings obtained at the end of the measurements revealed that the SCIT group showed significant improvements in functioning and subdomains of functioning compared to the control group. In this study investigating the effect of the SCIT intervention on occupational functioning, functioning was assessed using the FAST. The FAST scale evaluates individuals’ functionality levels across six key domains: autonomy, occupational functioning, cognitive functioning, financial issues, interpersonal relationships, and leisure activities. The individual analyses in the study reflected the improvements achieved through SCIT intervention in these areas, supported by subjective reports. For instance, participants who experienced difficulties fulfilling responsibilities at work, maintaining focus, or managing social relationships before therapy showed improvements in these areas following SCIT, which was reflected in decreased scores. Subjective accounts further supported these developments, highlighting that FAST is not only a numerical evaluation tool but also an instrument that captures the positive changes occurring in individuals’ lives. For example, one participant reported frequently procrastinating on tasks and encountering challenges in teamwork prior to therapy but stated that they took on a more active role and received positive feedback from colleagues after SCIT. This improvement was reflected in a reduction of their FAST score from 3 to 1. These findings emphasize that SCIT has a particularly significant impact on occupational functioning and interpersonal relationships. Significant differences were found in occupational and other areas of functioning in the SCIT group compared to the control group.

In previous studies, it has been reported that social functioning can be improved after the application of SCIT to patients with schizophrenic and psychotic disorders. Our findings for the improvement of functioning are consistent with other studies in the literature (Lahera et al., 2008; Wang et al., 2013; Roberts et al., 2014; Voutilainen et al., 2016; Zhang et al., 2019). In our study, in addition to occupational functioning on the FAST scale, functioning was assessed in the areas of autonomy, financial issues, interpersonal relationship, cognitive functioning and leisure time activities. Our findings in the areas of occupational functioning, cognitive functioning, interpersonal relationship, and leisure time activities showed that the SCIT group was superior to the control group. In some studies in the literature, it has been reported that patients with BD have impaired occupational functioning even in later life (Mehta et al., 2014; Samalin et al., 2016). It was observed that there was no significant difference in the autonomy and financial issues dimension among the general functionality areas of the participants. Since conditions such as domestic responsibilities and socio-economically independent life from the family come to the fore in the evaluation of autonomy, it can be said that it is an area where progress will be made in a long time (Weinstock and Miller, 2008). SCIT was found to be effective in identifying negative emotions and enhancing areas of functioning in BD patients. In the literature, there are very few studies examining the difficulties experienced by patients diagnosed with BD in work life other than clinical symptoms (Michalak et al., 2007; O’Donnell, 2017).

Our findings indicated that SCIT may play an effective role in improving the occupational functioning of BD-I patients. This suggests that SCIT may offer a valuable therapeutic approach for addressing the interpersonal and cognitive challenges faced by individuals with BD-I. The observed enhancements in social cognition among SCIT recipients are consistent with previous research highlighting the role of social cognitive deficits in BD-I and the potential benefits of targeted interventions in this domain (Voutilainen et al., 2016; Zhang et al., 2019). By focusing on enhancing skills such as perspective-taking, emotion recognition, and social problem-solving, SCIT may help individuals with BD-I navigate social interactions more effectively, thereby improving their interpersonal relationships and overall quality of life. Moreover, the improvements in occupational functioning following SCIT are particularly noteworthy, as occupational impairment is a common and significant outcome of BD-I. By addressing underlying social cognitive deficits and enhancing interpersonal skills, SCIT may empower individuals with BD-I to better manage workplace challenges, communicate effectively with colleagues and supervisors, and maintain stable employment, ultimately fostering greater independence and productivity.

Considering the socio-demographic data, it was observed that the factors of age, duration of illness and number of episodes related to the social cognitive level of patients diagnosed with bipolar disorder did not yield statistically significant results within the scope of this study, contrary to other studies in the literature (Bora et al., 2008; Budak et al., 2019). The socio-demographic and clinical characteristics of the experimental and control groups were compared in detail. It was found that there were no significant differences between the groups in factors such as age, gender, education level, and illness duration. This indicates that the groups were equivalent in their baseline conditions. This may be due to the small number of both intervention and control groups. On the other hand, it was determined that social cognition levels were affected by educational status. There are similar findings that the level of social cognition is related to educational level (Lahera et al., 2012; Rakoczy, 2022). The educational levels of the participants differ in terms of social cognition, and accordingly, it is understood that social cognition increases as the educational level increases.

It has been reported that medication adherence, which is another important part of treatment in bipolar disorder, is effective in reducing symptoms, but it is insufficient in the areas of prevention of recurrence of bipolar episodes, stigmatization dimension of the disorder and functionality (Kerr et al., 2003). Stigmatization is one of the most challenging areas related to mental illness in work life. Findings related to stigmatization revealed a significant difference only in the sub-dimension of resistance to stigmatization (Pischel and Felfe, 2023). In this sub- dimension, in which reverse scoring method was used in the scoring phase, it can be said that the fact that the participants can be together with people with the same diagnosis as themselves in group experiences helps them to strengthen against stigmatization. In another study, the effects of the feeling of stigmatization that started after being diagnosed with bipolar disorder on functionality were examined and it was concluded that stigmatization negatively affected socialization with the destruction it caused in self-esteem (Aydemir et al., 2013). In our study, according to the findings of the ISMI administered to the intervention and control groups before and after SCIT, a positive and significant difference was observed only in the sub-dimension of resistance to stigmatization. It was understood that the scores observed in the social withdrawal sub-dimension were also effective in the functionality of the participants. The resistance to stigmatization sub-dimension evaluates the individual’s resistance to stigmatization by reverse scoring. In this sub-dimension of the scale, an increase in resistance to stigmatization was observed compared to the pre-intervention period. The effect size of the resistance to stigmatization subscale was also found to be strong. Studying stigmatization and the internalized. Dimension at the same time can help this negative process to reach a manageable point (Perich et al., 2022; Post et al., 2018). The findings show that stigmatization is an area that needs to be studied in the long term, as no significance was observed in other sub-dimensions. From a clinical perspective, it is important to increase research to strengthen the areas of stigmatization and social cognition that make it difficult to integrate into work life in the treatment of mental illness.

The study has a number of limitations. First, the small sample size represents a significant constraint, as the structure of group therapy inherently requires a limited number of participants to maintain effective group dynamics and foster meaningful interpersonal interactions. Additionally, the strict inclusion and exclusion criteria further narrowed the pool of eligible participants, which in turn limits the generalizability of the findings. In addition to this, the lack of sessions that directly addressed patients’ emotions and stigmatization limited the study, according to the results of individual interviews with participants. The content that can be prepared for emotions such as anxiety, anger and paranoia, which are difficult to control, can contribute to the development of the emotional control that people with BD. Future adaptations of SCIT could incorporate supplementary sessions specifically tailored to address anxiety and stigma more directly. These sessions might include evidence-based approaches such as emotion regulation techniques and psychoeducational modules on stigma, thereby expanding the intervention’s focus to encompass these emotional dimensions. Such refinements have the potential to enhance the overall efficacy of SCIT by simultaneously targeting social cognition and emotional well-being in a more comprehensive manner.

As the control of emotions increases, it is expected that people diagnosed with BD will have less need for hospitalization and treatment costs will decrease. Second, the possible effects of mood stabilizers on cognitive performance between SCIT and control groups were not considered. Third, the study sample was small and the intervention was relatively short. Future research with larger, more diverse samples and longer follow-up periods is needed to further elucidate the long-term effects of SCIT on functioning and to explore the potential effects of treatment outcomes.

In conclusion, the findings of this study suggest that SCIT holds promise as an effective adjunctive intervention for improving social cognition and occupational functioning in individuals with BD-I. By addressing core deficits in social cognition and interpersonal skills, SCIT may help individuals with BD-I lead more fulfilling and functional lives. However, efforts to reduce stigma and promote social inclusion remain critical components of comprehensive treatment approaches for BD-I and other mental health conditions.

## 5 Conclusion

Our study demonstrates the promising efficacy of social cognition and interaction therapy (SCIT) as a complementary intervention in enhancing the social cognition and occupational functioning of individuals diagnosed with bipolar disorder type I (BD-I). At the end of an eight-week group intervention, significant improvements in clinical symptoms, occupational functioning and various other aspects of functioning were observed among participants receiving SCIT compared to those in the control group. These findings align with existing literature highlighting the positive impact of SCIT on social functioning in psychiatric populations, underscoring its potential as a valuable therapeutic approach for individuals with BD-I. Notably, our study adds to the growing body of evidence by revealing specific enhancements in occupational functioning, an area often significantly impaired in individuals with BD-I. The small sample size has been acknowledged as a limitation that restricts the generalizability of the findings, and the study has been identified as a pilot investigation. Future research with larger sample sizes has been recommended. Moreover, our research sheds light on the importance of addressing stigmatization, a pervasive challenge in mental health care, and highlights the potential of SCIT in bolstering resistance to stigmatization among individuals with BD-I. While our study focused on short-term outcomes, it underscores the need for future research with larger sample sizes and longer follow-up periods to comprehensively assess the lasting effects of SCIT on functionality and treatment outcomes. In sum, our findings support the integration of SCIT into comprehensive treatment approaches for BD-I, offering hope for improved social cognition, enhanced interpersonal skills, and ultimately, greater quality of life for individuals grappling with this complex disorder. However, we emphasize the ongoing importance of holistic interventions that address not only symptomatology but also broader psychosocial challenges, including stigma reduction and social inclusion, to optimize outcomes for individuals living with BD-I and other mental health conditions.

## Data Availability

The original contributions presented in the study are included in the article/supplementary material, further inquiries can be directed to the corresponding authors.
